# INDY—A New Link to Metabolic Regulation in Animals and Humans

**DOI:** 10.3389/fgene.2017.00066

**Published:** 2017-05-24

**Authors:** Blanka Rogina

**Affiliations:** Department of Genetics and Genome Sciences, Institute for Systems Genomics, School of Medicine, University of Connecticut Health Center, FarmingtonCT, United States

**Keywords:** *mIndy*, SLC13A5, aging, metabolism, longevity gene, calorie restriction, non-alcoholic fatty liver disease

## Abstract

The *Indy* (*I’m Not Dead Yet*) gene encodes the fly homolog of the mammalian SLC13A5 citrate transporter. Reduced expression of the *Indy* gene in flies and worms extends their longevity. INDY is expressed in the plasma membrane of metabolically active tissues. Decreased expression of *Indy* in worms, flies, mice, and rats alters metabolism in a manner similar to calorie restriction. Reducing INDY activity prevents weight gain in flies, worms, and mice, and counteracts the negative effects of age or a high fat diet on metabolism and insulin sensitivity. The metabolic effects of reducing INDY activity are the result of reduced cytoplasmic citrate. Citrate is a key metabolite and has a central role in energy status of the cell by effecting lipid and carbohydrate metabolism and energy production. Thereby newly described drugs that reduce INDY transporting activity increase insulin sensitivity and reduce hepatic lipid levels via its effect on hepatic citrate uptake. A recent report presented the first direct link between increased hepatic levels of human INDY, insulin resistance, and non-alcoholic fatty liver disease in obese humans. Similarly increased hepatic *mIndy* levels were observed in non-human primates fed on a high fat diet for 2 years. This effect is mediated via the stimulatory effect of the interleukin-6/Stat3 pathway on mINDY hepatic expression. These findings make INDY a potential and very promising target for the treatment of metabolic disorders in humans.

## INDY Reduction Affects Metabolism, Health, and Longevity

The *Indy* (*I’m Not Dead Yet*) gene encodes the fly homolog of the mammalian SLC13A5 transporter of the tricarboxylic acid (TCA) cycle intermediates (Rogina et al., 2000; Knauf et al., 2002, 2006). INDY is a member of the SLC13 protein family of Na^+^-coupled di- and tri-carboxylate/sulfate transporters in prokaryotes and eukaryotes (Bhutia et al., 2017). In flies, INDY mediates a cation independent and electroneutral high affinity bidirectional transport of the TCA intermediate across the plasma membrane (Knauf et al., 2002, 2006). Fly INDY has the highest substrate affinity for transporting citrate and lower affinities to other Krebs cycle intermediates such as succinate, malate, and fumarate (Knauf et al., 2002, 2006). INDY homologs in bacteria and mammals have the highest affinity for transporting citrate, and lower for other Krebs cycle intermediates but this transport is Na-dependent. The Na-citrate stoichiometry is 1:1 in bacteria and 4:1 for mINDY (Inoue et al., 2002a,b; Bhutia et al., 2017; von Loeffelholz et al., 2017).

Although all mammalian INDY transporters are Na^+^-dependent, the human mINDY is a high-capacity and low affinity transporter, while the rodent mINDY are low-capacity and high affinity transporters (Bhutia et al., 2017; von Loeffelholz et al., 2017).

**FIGURE 1:**
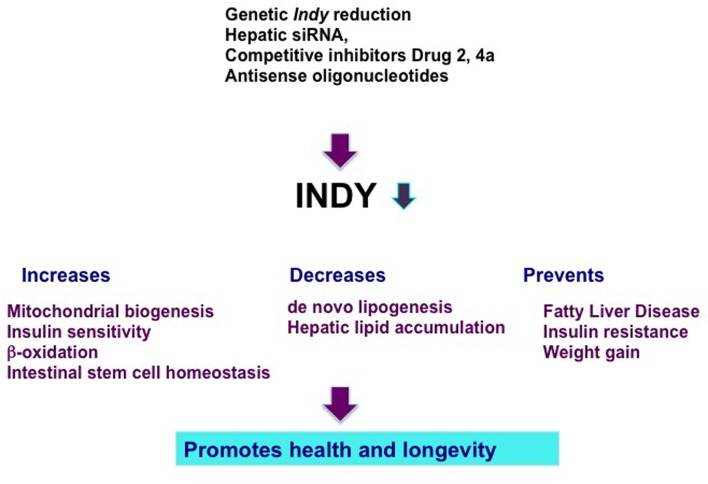
**Genetics and pharmacological manipulations that result in hepatic *Indy* reduction prevent negative effects of a high fat diet and promote health and longevity**. INDY reduction increases mitochondrial biogenesis, insulin sensitivity, β-oxidation, and preserves intestinal stem cell homeostasis. Reduced INDY decreases *de novo* lipogenesis and hepatic lipid accumulation. Decrease in INDY transporting activity prevents fatty liver disease, insulin resistance, and weight gain.

Reduced expression of the *Indy* gene in flies and worms extends longevity in all but one study (Rogina et al., 2000; Fei et al., 2003, 2004; Toivonen et al., 2007; Wang et al., 2009; Rogina and Helfand, 2013; Rogers and Rogina, 2014; Schwarz et al., 2015). INDY is expressed on the plasma membrane of metabolically active tissues. In flies INDY is predominantly expressed in the midgut, fat body, and oenocytes (fly liver) (Rogina et al., 2000; Knauf et al., 2002). In humans, *Indy* mRNA is mainly expressed in the liver, less in the brain and testis, while small levels of *Indy* mRNA expression were found in the kidneys, thymus, ovaries, adipose tissue, stomach, and colon (Inoue et al., 2002b; von Loeffelholz et al., 2017). Decreased expression of *Indy* in worms, flies, mice, and rats alters metabolism in a manner similar to calorie restriction (CR; Rogina et al., 2000; Fei et al., 2003; Bross et al., 2005; Neretti et al., 2009; Wang et al., 2009; Birkenfeld et al., 2011; Schwarz et al., 2015). This is supported by similar phenotypes found in CR wild type flies and in *Indy* flies that were kept on a high calorie diet. These *Indy* flies have lower lipid levels, increased mitochondrial biogenesis, increased spontaneous physical activity and a reduction in components of the insulin-signaling pathway activity (**Figure 1**; Neretti et al., 2009; Wang et al., 2009; Rogers and Rogina, 2014). *Indy* flies are protected from weight gain when aged on a high calorie diet (Wang et al., 2009). Under standard condition, heterozygous *Indy* flies do not experience any negative effects on health and have the same negative geotaxis, metabolic rate and maximal flight velocity (Marden et al., 2003). Furthermore, *Indy* heterozygous flies laid more eggs during their life compared to controls (Marden et al., 2003). However, under CR condition, *Indy* heterozygous flies have reduced fecundity due to lower energy resource caused by the effect of reduced *Indy* on metabolism (Marden et al., 2003). Consistently, CR does not further extend longevity of long-lived *Indy* heterozygous flies and shortens longevity of *Indy* homozygous flies (Wang et al., 2009).

Preservation of intestinal stem cell (ISC) homeostasis has a key role in maintaining normal midgut function and contributes to extended health and longevity in flies (Biteau et al., 2010). Changes in mitochondrial biogenesis found in the midgut of *Indy* flies, combined with increased antioxidant activity and reduced production of reactive oxygen species preserve ISC homeostasis and intestinal integrity in *Indy* flies. These changes maintain midgut function and mediate extended health and longevity of *Indy* flies (Rogers and Rogina, 2014).

Reduced activity of the *Indy* homologs in other organisms is associated with similar metabolic effects that mimic CR. siRNA mediated knockdown of Indy/CeNac2, the worm *Indy* homolog, results in worms that are smaller, have reduced lipid levels, and have extended longevity (Fei et al., 2004; Schwarz et al., 2015). *mIndy^-/-^* knockout mice are protected from the negative effects of aging or a high-fat diet on metabolism, which include hepatic fat accumulation, obesity, and insulin insensitivity (Birkenfeld et al., 2011). These mice have increased energy expenditure, reduced hepatic lipogenesis, increased mitochondrial biogenesis, and enhanced hepatic fatty acid (FA) oxidation (Birkenfeld et al., 2011). Increased liver accumulation of diacylglycerols (DAG) and ceramides have been linked to insulin resistance and development of type 2-diabetes (T2D) (Jornayvaz and Shulman, 2012). *mIndy^-/-^* mice have reduced DAG levels, which most likely contributes to their protection against insulin resistance. Whole-genome microarray studies comparing *mIndy^-/-^* and *mIndy^-/+^* revealed that transcriptional changes found in the liver of *mIndy^-/-^* mice are 80% identical to changes found in the liver of CR mice (Birkenfeld et al., 2011). All of these findings confer that INDY reduction creates a state similar to CR.

The metabolic effects of reduced INDY activity are a result of decreased cytoplasmic citrate levels. Citrate is converted to oxaloacetate and acetyl-CoA by ATP-citrate lyase. Acetyl-CoA is precursor for biosynthesis of triglycerides, FAs, low-density lipoproteins, and cholesterol. Citrate inhibits catabolism of glucose by inhibiting phosphofructokinase through allosteric modulation. Citrate also activates acetyl-CoA carboxylase, thereby affecting *de novo* lipogenesis. Thus, cytoplasmic citrate levels affect lipids and glucose metabolism, as well as energy production in mitochondria. Reduced *Indy* levels are associated with reduced ATP levels in *mIndy^-/-^* mice and in worms in which INDY levels are reduced by siIndy (Birkenfeld et al., 2011; Schwarz et al., 2015). The low ATP/ADP ratio activates AMPK, an energy sensor in the cells, which increases mitochondrial biogenesis by activating mitochondrial transcriptional co-activator peroxisome proliferator-activated receptor gamma coactivator 1-alpha (PGC-1α) (Guarente, 2008; Neretti et al., 2009; Birkenfeld et al., 2011; Rogers and Rogina, 2014; Schwarz et al., 2015). AMPK also increases insulin sensitivity, contributing to the beneficial effects of *Indy* reduction on glucose metabolism. When cytoplasmic citrate levels are high, FA β-oxidation is down regulated, while FA synthesis is upregulated. The opposite is found in *mIndy^-/-^* mice, in which low cytoplasmic citrate levels result in reduced FA synthesis, while FA β-oxidation and insulin sensitivity is increased (Birkenfeld et al., 2011).

## Regulation of *mIndy* Transcription

**FIGURE 2:**
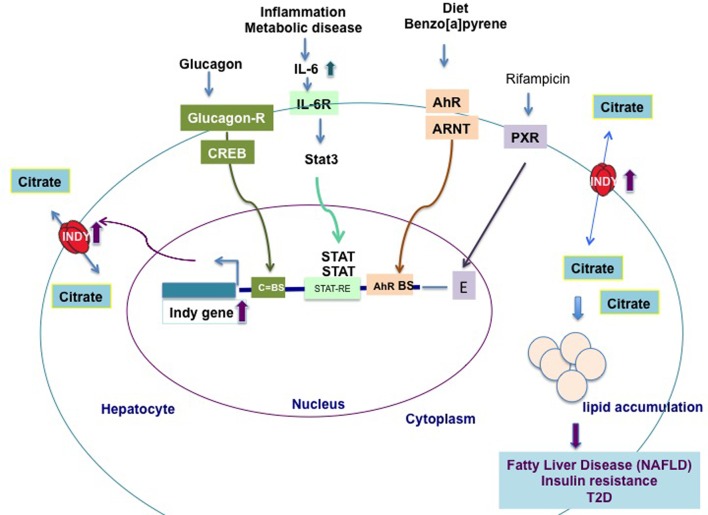
**Transcriptional regulation of hepatic *mIndy* mRNA levels in model organisms and humans**. Hepatic *mIndy* expression levels are upregulated by glucagon released during early starvation via a cAMP and cAMP-responsive-element binding-protein (CREB)-dependent mechanism. A CREB binding site (C-BS) was identified in the promoter region of mINDY. Increased levels of interleukin-6 (IL-6) associated with inflammation and metabolic disease, activate transcription of hepatic *mIndy* via the signal transducer and activator of transcription 3 (STAT-3) signaling pathway. Diet and benzo[a]pyrene activate aryl hydrocarbon receptor (AhR) and its heterodimerization to AhR nuclear translocator (ARNT). In nucleus AhR activates transcription of *mIndy* via binding to potential binding site (BS) in the mIndy promoter (Neuschafer-Rube et al., 2015). Rifampicin activates the pregnane X receptor (PXR), which increases *mIndy* transcription by activation of two enhancer modules located upstream of the *mIndy* (*SLC13A5*) transcriptional start site (Li et al., 2015). Increased INDY levels result in higher citrate uptake and its incorporation in lipids, leading to non-alcoholic fatty liver disease (NAFLD), insulin resistance and type 2-diabetes (T2D).

The levels of fly INDY are affected by age and by caloric content of the food. Aging flies on a standard diet, young flies on a high calories diet, or young flies treated with paraquat (creating oxidative stress), have increased *Indy* mRNA and protein levels in the midgut (Rogers and Rogina, 2014). In contrast, flies aged on a CR diet have *Indy* mRNA reduced to 50% of the levels found in controls (Wang et al., 2009; Rogers and Rogina, 2014). Recent work showed that *mIndy* levels in primary rat hepatocytes are regulated by glucagon released during early starvation (Neuschafer-Rube et al., 2015). Glucagon binds to the CREB (cAMP-dependent and cAMP-responsive element protein)-dependent binding site in the promoter region of *mIndy* and transiently increases *mIndy* expression (**Figure 2**) (Neuschafer-Rube et al., 2014). *In vivo* studies shown increased hepatic *mIndy* levels in high-fat-diet-streptozotocin diabetic rats, in which CREB is constitutively active.

Additional findings link increased INDY to lipid metabolism. SLC13A5 has been identified as a novel transcriptional target of the pregnane X receptor (PXR) (Li et al., 2015). The PXR has been linked to lipid metabolism and energy homeostasis. Rifampicin activates the PXR, which increases *mIndy* transcription by activation of two enhancer modules located upstream of the *mIndy* (*SLC13A5*) transcriptional start site (Li et al., 2015). Rifampicin-induced PXR activation induces SLC13A5 mRNA and protein levels, and the lipid content in human primary hepatocytes, while SLC13A5 knockdown expression significantly reduces the lipid content in HepG2 cells (Li et al., 2015). These data suggest a role of increased INDY levels in drug-induced steatosis in human liver.

Energy-rich diet and benzo[a]pyrene activate aryl hydrocarbon receptor (AhR). AhR heterodimerization to AhR nuclear translocator (ARNT) allows its translocation to nucleus and activation of *mIndy* transcription via binding to potential binding site in the mIndy promoter. AhR putative binding site has been identified in the promoter region of both rat and human mIndy (Neuschafer-Rube et al., 2015). Activation of the AhR leads to fatty liver disease.

Patients undergoing Li^+^ treatment have dyslipidemia and gain body weight. The stimulatory effects of Li^+^ on the transporting activity of mINDY have been described, suggesting possible clinically relevant connection between increased INDY activity and obesity in humans (Inoue et al., 2003). Taken together, increased hINDY levels may contribute to clinically relevant hepatic metabolic dysfunction associated with T2D.

## Increased INDY Levels are Linked to Non-Alcoholic Fatty Liver Disease in Humans

Two recent reports linked increased *mIndy* levels to non-alcoholic fatty liver disease (NAFLD) in an experimental mice model of NAFLD and human patients with NAFLD (Brachs et al., 2016; von Loeffelholz et al., 2017). NAFLD is associated with development of insulin resistance, T2D and liver cirrhosis (Farrell and Larter, 2006; Postic and Girard, 2008). NAFLD results from sedentary life style, increased energy uptake, reduced FA oxidation, and possible altered gut microbiota (Postic and Girard, 2008). Diet-induced NAFLD in adult C57BL/6J mice fed a western diet was prevented by weekly injection of liver specific siRNA against mINDY, which suppressed 60% of liver *mIndy* levels after 8 weeks (Brachs et al., 2016). These mice have improved hepatic insulin sensitivity, reduced hepatic lipid accumulation, and are protected against diet-induced lipid-steatosis. Notably, activation of the AhR leads to fatty liver disease and induces *mIndy* expression in rat hepatocytes, **Figure 2** (Neuschafer-Rube et al., 2015).

The first direct link between increased hepatic mIndy levels and lipid steatosis in human NAFLD patients was reported by von Loeffelholz et al. (2017). The authors showed that, in lean individuals, low levels of hepatic fat are associated with decreased mIndy expression, while in individuals with an increased body mass index and a high liver fat content mINDY levels are increased. Similarly, mINDY levels in the liver are increased in non-human primates aged on a high-fat or high-sucrose diet and in mice on a high-fat or a high fat-nonalcoholic steatohepatitis (NASH) inducing (low methionine, choline-deficient) diet. Liver microarray studies performed in samples from NAFLD patients and controls revealed an association between changes in whole-genome transcriptional levels in NAFLD liver samples and an increase in the expression of genes involved in lipid synthesis, metabolism, and immunological processes (von Loeffelholz et al., 2017). An increase in interleukin-6 (IL-6) levels activates mIndy transcription via binding to the IL-6-receptor, which is associated with phosphorylation, activation of the transcription factor Stat3, and transfer of the latter to the nucleus, where it binds to the putative STAT-responsive element (STAT-RE) binding site in the *mIndy* promoter. The *in vivo* activation of the IL-6/Stat3 pathway increases mIndy expression and thereby citrate uptake and hepatic lipogenesis (**Figure 2**). In other *in vivo* studies, the administration of IL-6 for 14 days increased citrate uptake and FA synthesis in the livers of *mIndy^+/+^* mice, by increasing *mIndy* mRNA levels (von Loeffelholz et al., 2017). This result confirmed the link between IL-6 and increased mIndy. The IL-6 mediated increase in mIndy citrate activity was confirmed by the lack of citrate uptake in hepatocytes isolated from *mIndy^-/-^* knockout mice, consistent with the link between mIndy and IL-6 (von Loeffelholz et al., 2017).

## *mIndy* is a Potential Therapeutic Target for Treating Hepatic Steatosis and Insulin Resistance

Considering the beneficial effects of the reduced transporting activity of INDY on metabolism and health, we and others have suggested mIndy (SLC13A5) as a target for the treatment of metabolic disorders (**Figure 1**; Rogina et al., 2000; Birkenfeld et al., 2011; Frankel and Rogina, 2012; Willmes and Birkenfeld, 2013; Rogers and Rogina, 2015). Several recent reports have described successful efforts to target the transporting activity of INDY and novel compounds have been identified that demonstrate the therapeutic potential of mINDY inhibitors in the treatment of NAFLD and insulin resistance. Temporal administrations of inducible hepatic 2′-*O*-methoxyethyl chimeric antisense oligonucleotides to knockdown *mIndy* levels in rats alleviated the negative effects of a high fat diet (HFD) and prevented diet-induced hepatic steatosis and hepatic insulin resistance without affecting rat body weight (**Figure 1**; Pesta et al., 2015). These rats had lower serum triglyceride levels and improved hepatic insulin sensitivity. Several competitive stereo-specific inhibitors of mIndy transporting activity have been identified (Huard et al., 2015, 2016). Small dicarboxylate molecules selectively inhibit mIndy citrate uptake *in vitro* in human hepatocytes and hepatic citrate uptake *in vivo* in mice. Compound 2 is a competitive inhibitor of mIndy that results in lower hepatic lipid levels, reduced plasma glucose levels, and a reversal of glucose intolerance in HFD-fed mice (Huard et al., 2015). Compound 4a is a potent mIndy inhibitor (Huard et al., 2016). Rodents treated with compound 4a had reduced citrate uptake in the liver, kidneys, and testis, which resulted in modest improvements in glucose metabolism (Huard et al., 2016).

## Mutations in *mIndy* (*SLC13A5*) Lead to Autosomal-Recessive Epileptic Encephalopathy with Neonatal Seizures

Mutations in human *mIndy* cause autosomal-recessive epileptic encephalopathy with seizures during the first days of life and lead to developmental delays (Thevenon et al., 2014). *mIndy* encodes the only known neuronal plasma membrane Na-dependent citrate transporter, and gene mutations typically involve the sodium-binding domains of the respective protein. The key steps in mINDY-mediated citrate transport across the plasma membrane depend on conformational changes associated with Na-binding to critical residues within two mINDY domains. Thus, it has been speculated that the severe phenotype induced by human *mIndy* mutations is most likely due to the inability of mutated INDY to bind the sodium required for transporting citrate across the plasma membrane (Thevenon et al., 2014). *In vitro* studies showed that COS-7 cells transiently transfected with *mIndy* mutant genes lack citrate transport capacity (Klotz et al., 2016). Recently, the European Consortium identified additional *mIndy* mutations that give rise to premature stop codons or amino acid substitutions in mINDY and result in the absence of citrate transport (Hardies et al., 2015; Weeke et al., 2017). These *mIndy* mutations are also associated with neonatal epilepsy, developmental delays, and variable cognitive impairment. Several patients have dental hypoplasia or hypodontia (Hardies et al., 2015). Neuroimaging findings of eight full-term infants with *mIndy* mutations showed punctate white-matter lesions (Weeke et al., 2017). Once in the cytoplasm, citrate is used for lipid, cholesterol, glucose, and glutamate synthesis. Malate derived from citrate is transported into mitochondria, where it is used for energy production. Thus, epilepsy may be due to disrupted energy production and altered metabolism in brain cells, an imbalance in GABA and glutamate production, or a reduced inhibition of excitatory *N*-methyl-D-aspartate (NMDA) receptors (Hardies et al., 2015). However, the pathophysiology of *mIndy* mutations and the mechanism underlying severe phenotypes are unknown. Notably, *mIndy^-/-^* mice or mice treated with small mIndy competitive inhibitors did not develop seizures.

## Summary and Concluding Thoughts

Reduction of *Indy* gene activity in flies and worms extends their health and longevity (Rogers and Rogina, 2015). Genetically reduced INDY expression has beneficial effects on metabolism and prevents diet-induced obesity in flies and mice, suggesting INDY as a target in the treatment of metabolic disorders in humans (Wang et al., 2009; Birkenfeld et al., 2011; Rogers and Rogina, 2015). This potential is supported by several findings. For example, reduced plasma insulin and plasma triglyceride levels as well as an improvement of hepatic steatosis were observed in HFD-fed mice and rats in which liver-specific knockdown of *Indy* was accomplished using a low-dose of a chimeric anti-sense oligonucleotide (Pesta et al., 2015). Additional pharmacological treatments were recently described, such as competitive inhibitors of INDY transport activity that improve insulin sensitivity in rats and mice *in vivo* and in cultured human cells (Huard et al., 2015, 2016).

By contrast, high levels of INDY are associated with negative effects on metabolism and health, while *mIndy* gene mutations cause autosomal-recessive epileptic encephalopathy in newborns as well as developmental delays (Thevenon et al., 2014; von Loeffelholz et al., 2017). Further studies are needed to reveal the mechanisms that lead to this severe phenotype in patients. Recently, increased hepatic levels of *mINDY* were linked to insulin resistance and NAFLD in obese humans (von Loeffelholz et al., 2017). These findings illustrate both the relevance of the *mIndy* gene to human health and a highly conserved role for INDY in the metabolism of a broad range of species. Thus, mIndy has emerged as a novel target for the treatment of age- and diet-associated metabolic syndrome, NAFLD, and T2D. The further development of mIndy inhibitors may additionally provide effective interventions targeting the debilitating health effects that are often associated with aging and will thereby allow a healthier life.

## Author Contributions

The author confirms being the sole contributor of this work and approved it for publication.

## Conflict of Interest Statement

The author declares that she is a co-owner of the INDY US Patent (#7,118,873).
